# Sacral Fatigue Fracture in an Amateur Soccer Player

**DOI:** 10.1155/2013/985310

**Published:** 2013-05-23

**Authors:** Georgios Tzoanos, Nikolaos Tsavalas, Nikolaos Manidakis, Apostolos Karantanas

**Affiliations:** ^1^Department of Orthopaedic Surgery and Traumatology, University Hospital, Stavrakia-Voutes, Heraklion 71110, Crete, Greece; ^2^Department of Medical Imaging, University Hospital, Heraklion 71110, Crete, Greece

## Abstract

Sacral fatigue fractures represent a frequently overlooked cause of low-back and buttock pain in athletes. A high index of clinical suspicion and MRI utilization can provide the accurate diagnosis. A 38-year-old male amateur, midfielder, soccer player presented to our department with aggravating right buttock pain during the previous month, following an increase in training intensity and frequency on an artificial turf field. A point of maximal tenderness was demonstrated over the area of the right sacroiliac joint. No radiographic abnormalities were observed. MRI of the pelvis revealed the presence of a stress fracture in the right sacral ala. The patient underwent conservative treatment and resumed playing soccer 12 weeks later, with no residual or recurrent clinical complaints. Apart from the recent change in training regimen, decreased shock absorption related to the physical properties of old generation artificial turf may have also been involved in this case.

## 1. Introduction

 Stress fracture incidence among athletes is estimated at 2–4% [1, 2]. Sacral fatigue fractures represent an unusual cause of low-back and buttock pain in competitive sports population. Associated symptoms can be falsely attributed to musculotendinous, ligamentous, or discogenic causes [3]. Furthermore, conventional radiographs are often normal [4] rendering sacral fatigue fractures an underdiagnosed entity [3]. Magnetic resonance imaging (MRI) is indicated when a stress fracture is suspected clinically [5–7]. We present the first case of an amateur soccer player with a sacral fatigue fracture.

## 2. Case Presentation

A 38-year-old male amateur, midfielder, soccer player was referred to the orthopaedic department of our institution due to persistent, aggravating right buttock pain during the previous month. He reported an insidious onset of discomfort as he had increased intensity and frequency of training, to participate in the upcoming season of local soccer league. Each practice session was based on plyometrics, strength, balance, jumping, plant and cut, bounding, and running exercises performed on a second-generation artificial turf field. Pain was exacerbated by activity and relieved with rest, worsening over time. No paresthesia or weakness to the leg was experienced. His past medical history was unremarkable, with no report of any recent or older injury. 

**Figure 1:**
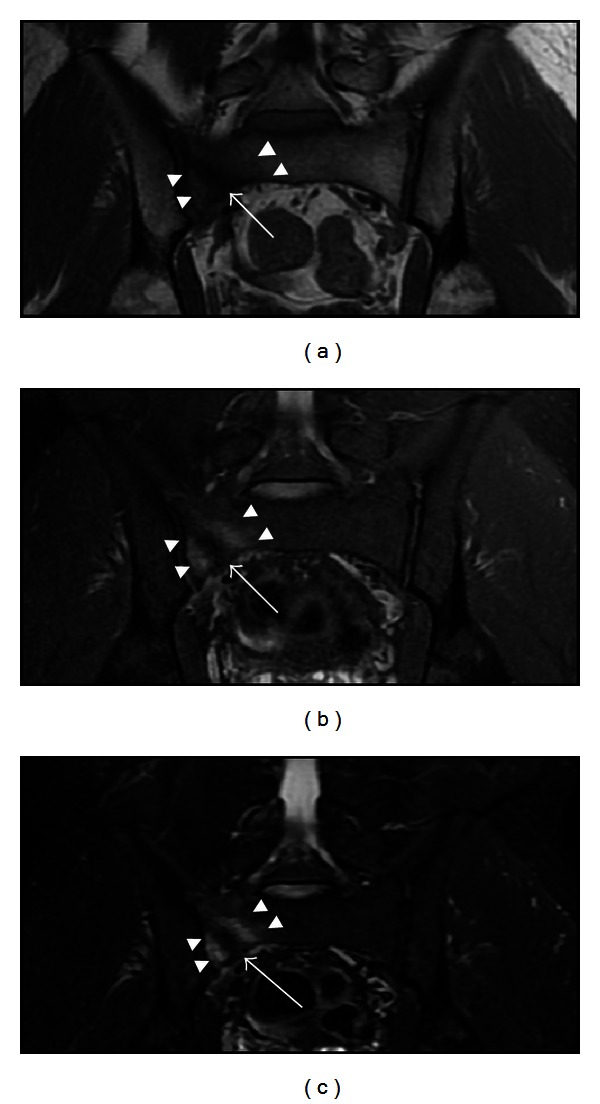
The coronal T1-w (a), STIR (b) and fat-suppressed T2-w (c) MR images of the pelvis depict an area of bone marrow edema in the right sacral ala (arrowheads) surrounding a low signal intensity obliquely oriented vertical fracture line (arrow).

On physical examination, a point of maximal tenderness was revealed above the midportion of his right buttock, in the area of the ipsilateral sacroiliac joint. Tenderness was also found in the right pubic ramus with extension to the pubic symphysis. Manual motor tests of both lower extremities demonstrated 5+/5 strength, with full range of joint motion. Sensation to light touch and pinprick were intact. An anteroposterior radiograph of the pelvis was performed; the findings of which were interpreted as normal. Subsequent MRI of the pelvis revealed the presence of a stress fracture in the right sacral ala (Figure 1).

A diagnosis of a sacral fatigue fracture was established on the basis of both clinical and imaging data. According to our suggestions, the patient temporarily withdrew from sports activities, received analgesics, and underwent a six-week physiotherapy program focused on stretching of the posterior leg muscles, abdominal muscle strengthening, and core stabilization maneuvers. Running and soccer-specific training were then commenced with a gradual increase in the amount of practice in a weekly schedule. The patient resumed playing soccer 12 weeks later, with no residual or recurrent clinical complaints.

## 3. Discussion

 Stress fractures are particularly uncommon in elite male soccer players, accounting for 0,5% of all injuries. Repetitive stress, especially during the preseason training period, and rapid changes of load between seasons are considered important risk factors. The fifth metatarsal bone is affected in most cases [8]. 

 Sacral stress fractures are rare injuries among athletes, mainly seen in young female long-distance runners, and are considered to be an uncommon source of low-back pain [9–13]. Stress fractures were initially described in the metatarsals of military recruits after long marches. Sacral involvement was first observed in 1989 [14, 15]. Cases have been reported in male and female runners [3, 11, 16, 17], basketball [18], volleyball [12], tennis [2], and hockey players [13, 14], children and adolescents [19, 20], pregnant and postpartal women [21–23], and military recruits [15] and after lumbosacral arthrodesis [24, 25]. 

 Stress fractures are classified into insufficiency and fatigue types [6, 7]. In the sacrum, insufficiency fractures are far more prevalent [17]. Fatigue fractures typically result from chronic repetitive abnormal stress applied to healthy bone [14]. A vertical concentration of cycling overloading in the sacral bone combined with impaired shock absorption due to associated muscle fatigue has been postulated as a potential mechanism in regard to sacral fatigue fracture pathogenesis [3, 10]. High-intensity training and/or rapid changes in training regimen are common predisposing factors [3, 12, 17, 26]. Leg length discrepancy [10, 12, 17], footwear more than 6 months old [12, 26], poor training surface [17, 26], nutritional deficiencies, and the female athlete triad [3, 10, 17, 27] have also been implicated as risk factors, among others. 

 In our case, a recent increase in training intensity and frequency is the “obvious culprit.” Additionally, the training surface may have played a contributing role. The relatively high surface stiffness and friction characterizing first- and second-generation artificial turf fields may lead to poor impact attenuation. Associated decreased shock absorption has been implicated in lower body overuse injuries [28].

 A high index of clinical suspicion is required in the diagnosis of stress fractures. Associated soft tissue injury may mimic and conceal the underlying osseous pathology [9]. Sacral stress fracture patients usually present with unilateral low-back, buttock, or hip pain of insidious onset. A recent history of acute trauma is typically absent. Pain is often aggravated during weight-bearing activities [14, 16]. Associated ipsilateral sciatic nerve radiculopathy has been reported, although rarely [21, 29].

 Due to its relatively low cost, wide availability, and speed, radiography remains the first-line imaging examination of patients with suspected stress fractures. However, low-sensitivity issues may initially lead to normal radiographic findings. In such cases, more advanced imaging should be utilized. MRI can be of great value in stress fracture evaluation. Extensive bone marrow edema, depicted as a sizeable intraosseous area of low signal intensity on T1-w and corresponding high signal intensity on T2-w images, surrounding a distinct fracture line, which is of low signal intensity on all MR sequences, is consistent with the diagnosis of a stress fracture in the right clinical context [3–5, 7]. Sacral stress fractures are typically unilateral, involving the superior aspect of the sacral ala and assuming a vertical or oblique orientation, usually parallel to the sacroiliac joints [3, 13, 14]. In case of equivocal or nonspecific MRI findings, additional CT evaluation can reveal the fracture.

 Most sacral fatigue fractures are stable and heal with relative rest for about 4–6 weeks. Pain control with analgesics and physiotherapy are often recommended. A gradual return to sports activities after complete symptom resolution is of utmost importance and should be overemphasized to young athletes, as they are eager to engage in strenuous training too early in the recovery phase [2, 5, 10, 12, 19, 27]. Proper conservative treatment, following an early diagnosis, has proved adequate for an effective short-term rehabilitation in the majority of patients [5, 6]. Follow-up imaging and additional rest are indicated in refractory cases [16].

 To the best of our knowledge, this is the first reported case of a sacral fatigue fracture in an amateur soccer player. Apart from the recent change in training regimen, decreased shock absorption related to the physical properties of old generation artificial turf may have also been involved in our patient.
